# Fall, Fracture, and Two Episodes of Euglycemic Diabetic Ketoacidosis

**DOI:** 10.7759/cureus.25788

**Published:** 2022-06-09

**Authors:** Waiz Wasey, Sarah Hutchings, Anastasia Dufner, Dorathy Okon, Sharefi Saleh

**Affiliations:** 1 Family and Community Medicine, Southern Illinois University School of Medicine, Springfield, USA; 2 Family Medicine, Southern Illinois University School of Medicine, Springfield, USA; 3 Family Medicine, Ruth Temple Clinic, Los Angeles, USA

**Keywords:** anion gap metabolic acidosis, sodium-glucose cotransporter-2 (sglt2) inhibitors, euglycemic, diabetic ketoacidosis (dka), diabetes type 2

## Abstract

Diabetic ketoacidosis (DKA) is a serious diabetic complication that is characterized by hyperglycemia, metabolic acidosis, and ketosis. A subset of DKA patients may present with blood glucose levels <250 mg/dL which may delay the diagnosis. This subset is referred to as euglycemic DKA (euDKA). It is generally seen in pregnancy, prolonged fasting, and the use of sodium-glucose cotransporter 2 (SGLT2) inhibitors. The recent rise in the use of SGLT2 inhibitors to treat diabetes has increased the incidence of euDKA. We present the case of a 60-year-old female on SGLT2 inhibitors who presented after a ground-level fall and was not diagnosed with euDKA until the next morning. This case was further complicated by another episode of euDKA during the same admission, suggesting that euDKA is possible even after holding the SGLT2 inhibitors for a few days.

## Introduction

Diabetic ketoacidosis (DKA) is a potentially life-threatening complication of diabetes mellitus. It is characterized by a triad of hyperglycemia, metabolic acidosis, and ketosis [1]. A subset of DKA patients may have normoglycemia (blood glucose <250 mg/dL) which may mislead the clinician, resulting in a delayed diagnosis. The incidence of euglycemic DKA (euDKA) has increased significantly after the introduction of sodium-glucose cotransporter 2 (SGLT2) inhibitors [2]. Other causes of euDKA include pregnancy, prolonged fasting, heavy alcohol use, liver cirrhosis, and cocaine abuse.

Symptoms of DKA include polyuria and polydipsia secondary to hyperglycemia. Other symptoms include dry mouth, headache, tachypnea, nausea, vomiting, fruity mouth breath, and fatigue. Dehydration and hyperglycemia are minor in euDKA; hence, the class symptoms of polyuria and polydipsia may be absent as well [3]. However, they may present with weakness, anorexia, and tachypnea.

Here, we present a case of a middle-aged female who was admitted after a fall secondary to weakness. Due to normoglycemia, she had a delayed diagnosis of euDKA. The offending agent, SGLT2 inhibitors, were held at admission, and the patient was treated with insulin only for her diabetes during the hospitalization. The fall led to a humeral fracture that was surgically corrected. The stress from surgery led to another episode of euDKA, even though SGLT2 inhibitors were held five days prior to the second episode.

## Case presentation

**Figure 1:**
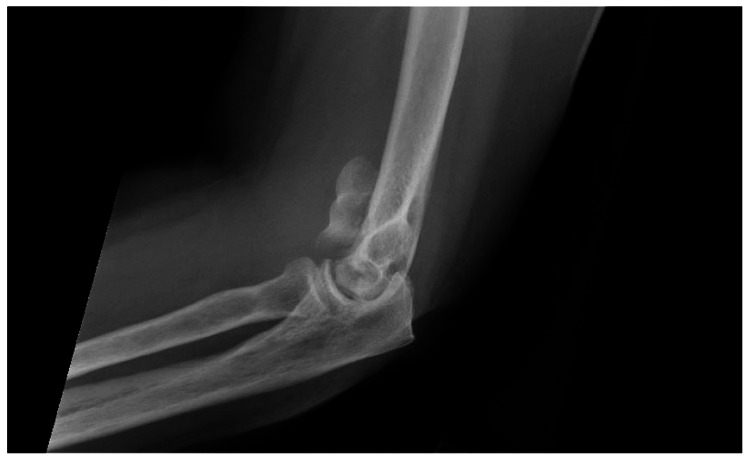
Comminuted intra-articular distal humerus fracture of the left upper extremity.

A 60-year-old female was brought to the Emergency Room (ER) after a ground-level fall that led to a comminuted intra-articular distal humerus fracture of the left upper extremity (Figure 1). She was overnight admitted to the orthopedic department, and the medical team was consulted for preoperative clearance the next day.

The patient’s medical history included heart failure with reduced ejection fraction (HFrEF), hypertension, type 2 diabetes, anxiety, and acid reflux. She was on aspirin, atorvastatin, lisinopril, metoprolol, torsemide, empagliflozin, insulin, metformin, gabapentin, sertraline, and pantoprazole. During the clinical interview, the patient reported feeling weak. Her admission lab results are presented in Table 1.

**Table 1:** Lab test findings on admission.

Lab test	Value	Reference
Hemoglobin	12.7	12–16 g/dL
Red blood cell count	4.95	4.2–5.4 k/cumm
White blood cell count	10.4	3.4–9.4 k/cumm
Platelets	211	140–410 k/cumm
Sodium	135	136–145 mmol/L
Potassium	4.2	3.5–5.1 mmol/L
Bicarbonate level	11	21–31 mmol/L
Anion gap	21	8–16
Creatinine	0.6	0.6–1.3 mg/dL
Glucose	228	70–105 mg/dL
Calcium	8.6	8.6–10.3 mg/dL
Urine analysis	Unremarkable	
Urine toxicology	Negative for cocaine	
Chest X-ray	Negative for consolidations	

Based on the patient’s presentation, an increased anion gap, and low bicarbonate, beta-hydroxybutyrate level was ordered. The level was elevated at 3.2 mmol/L (reference: 0-0.3). Blood gas analysis showed mild acidosis with a pH of 7.30.

She was diagnosed with DKA and transferred to the intensive care unit (ICU) for treatment. Once the gap was closed and acidosis had resolved, the patient went for open reduction internal fixation of the left distal humerus. During the admission, her SGLT2 inhibitor was held and diabetes was only treated with insulin.

Laboratory tests the next morning (day five of admission) showed an increased anion gap again. The rest of the values are presented in Table 2.

**Table 2:** Lab test findings on day five.

Lab test	Value	Reference
Sodium	133	136–145 mmol/L
Potassium	3.5	3.5–5.1 mmol/L
Bicarbonate level	12	21–31 mmol/L
Anion gap	19	8–16
Creatinine	0.5	0.6–1.3 mg/dL
Glucose	160	70–105 mg/dL
Calcium	8.1	8.6–10.3 mg/dL

A repeat beta-hydroxybutyrate level showed an increased level of 8.2 mmol/L. The patient was again successfully treated with the DKA protocol. Prior to discharge, she had stable lab values and closed anion gap for two days.

## Discussion

DKA is a serious complication of diabetes mellitus and often an endocrinological emergency. It is characterized by a triad of hyperglycemia, metabolic acidosis, and ketosis [1]. A number of DKA patients may have normoglycemia (blood glucose <250 mg/dL) on presentation, which may mislead the clinician leading to a delayed diagnosis. The incidence of euDKA has increased significantly after the introduction of SGLT2 inhibitors [2].

**Figure 2:**
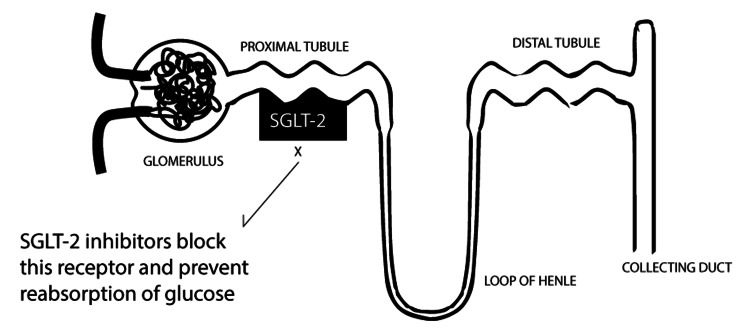
SGLT2 inhibitor mechanism of action of the renal tubule. Image credits: Waiz Wasey, MD. SGLT2: sodium-glucose cotransporter 2

SGLT2 inhibitors are a recently added group of medication used to treat type 2 diabetes and have been found to be protective against adverse cardiovascular incidents [4]. They have also been found to delay the progression of chronic kidney disease in diabetes [5]. The medicine works by blocking the SGLT2 cotransporter in the proximal renal tubule, which is responsible for the reabsorption of glucose. This leads to glucosuria and hence low blood glucose levels (Figure 2).

Other causes of euDKA include pregnancy, prolonged fasting, heavy alcohol use, liver cirrhosis, and cocaine abuse.

The majority of the reported euDKA cases appear to be in middle-aged women with a history of type 2 diabetes who have been on SGLT2 inhibitors for at least three months. The common presentations of these patients were fatigue, abdominal pain, reduced food intake, nausea, and vomiting [6]. The half-life of empagliflozin, the SGLT2 inhibitor, our patient was on is 12 hours and most of the drug is cleared within a few days [7].

Our patient presented to the ER with a ground-level fall which she reported was due to weakness. There is a possibility that the fall was due to the euDKA because weakness was the first symptom. In the ER, the patient went undiagnosed with DKA and was admitted to the floor for surgery. During the medical preoperative evaluation, a suspicion of euDKA led to the diagnosis. However, the diagnosis was delayed for at least 12 hours from admission. DKA is a serious medical emergency that may lead to morbidity or loss of life if untreated. Fortunately, our patient had mild acidosis and was treated without complications.

Even though her SGLT2 inhibitor was stopped from the day of admission, the stress from surgery most likely led to another episode of DKA, specifically euDKA. Our literature search did not find a case with two episodes of euDKA during the same admission. The second incidence occurred five days after stopping the SGLT2 inhibitor. As mentioned earlier, empagliflozin may take a few days to be completely cleared from the body. The stress from the surgery with the lingering effects of SGLT2 caused another episode of euDKA.

## Conclusions

EuDKA is a complication of diabetes, which, unlike DKA, does not present with hyperglycemia. The lower blood glucose levels mislead clinicians in most cases, often delaying diagnosis and treatment. Increased use of SGLT2 inhibitors has led to an increase in euDKA incidence. Our case was complicated by the second incident of euDKA despite stopping the medication, which suggests the effects of SGLT2 in glucose reabsorption may continue for a few days after cessation of the medication. Any diabetic patient on SGLT2 inhibitors should be suspected to have euDKA based on anion gap, bicarbonate levels, and acidosis unless proven otherwise.
